# Electron and Proton Flux for Carbon Dioxide Reduction in *Methanosarcina barkeri* During Direct Interspecies Electron Transfer

**DOI:** 10.3389/fmicb.2018.03109

**Published:** 2018-12-13

**Authors:** Dawn E. Holmes, Amelia-Elena Rotaru, Toshiyuki Ueki, Pravin M. Shrestha, James G. Ferry, Derek R. Lovley

**Affiliations:** ^1^Department of Microbiology, University of Massachusetts, Amherst, MA, United States; ^2^Department of Physical and Biological Sciences, Western New England University, Springfield, MA, United States; ^3^Department of Biology, University of Southern Denmark, Odense, Denmark; ^4^Assembly Biosciences, San Francisco, CA, United States; ^5^Department of Biochemistry and Molecular Biology, Pennsylvania State University, University Park, PA, United States

**Keywords:** syntrophy, methanogenesis, F_420_ dehydrogenase, heterodisulfide reductase, transcriptomics

## Abstract

Direct interspecies electron transfer (DIET) is important in diverse methanogenic environments, but how methanogens participate in DIET is poorly understood. Therefore, the transcriptome of *Methanosarcina barkeri* grown via DIET in co-culture with *Geobacter metallireducens* was compared with its transcriptome when grown via H_2_ interspecies transfer (HIT) with *Pelobacter carbinolicus*. Notably, transcripts for the F_420_H_2_ dehydrogenase, Fpo, and the heterodisulfide reductase, HdrABC, were more abundant during growth on DIET. A model for CO_2_ reduction was developed from these results in which electrons delivered to methanophenazine in the cell membrane are transferred to Fpo. The external proton gradient necessary to drive the otherwise thermodynamically unfavorable reverse electron transport for Fpo-catalyzed F_420_ reduction is derived from protons released from *G. metallireducens* metabolism. Reduced F_420_ is a direct electron donor in the carbon dioxide reduction pathway and also serves as the electron donor for the proposed HdrABC-catalyzed electron bifurcation reaction in which reduced ferredoxin (also required for carbon dioxide reduction) is generated with simultaneous reduction of CoM-S-S-CoB. Expression of genes for putative redox-active proteins predicted to be localized on the outer cell surface was higher during growth on DIET, but further analysis will be required to identify the electron transfer route to methanophenazine. The results indicate that the pathways for electron and proton flux for CO_2_ reduction during DIET are substantially different than for HIT and suggest that gene expression patterns may also be useful for determining whether *Methanosarcina* are directly accepting electrons from other extracellular electron donors, such as corroding metals or electrodes.

## Introduction

The mechanisms by which methanogens conserve energy to support growth during direct interspecies electron transfer (DIET) are of interest because it is becoming increasingly apparent that DIET may be an important alternative to hydrogen interspecies transfer (HIT) for methane production in anaerobic digesters as well as methanogenic soils and sediments (Shrestha and Rotaru, 2014; Dubé and Guiot, 2015; Cheng and Call, 2016; Lovley, 2017c). A better understanding of DIET could help with the development of molecular approaches that can be used to detect DIET in methanogenic environments (Rotaru et al., 2014b; Holmes et al., 2017) and might lead to new approaches for promoting DIET to accelerate and stabilize anaerobic digestion (Cheng and Call, 2016; Barua and Dhar, 2017; Lovley, 2017b,c; Baek et al., 2018; Park et al., 2018).

Physiological studies of DIET require defined co-cultures. *Geobacter metallireducens* is an environmentally relevant pure culture model for electron-donating partners for DIET because *Geobacter* species function as the electron-donating partner in important methanogenic environments such as anaerobic digesters (Morita et al., 2011; Rotaru et al., 2014b) and terrestrial wetlands (Holmes et al., 2017). Studies with defined co-cultures in which *G. metallireducens* was the electron-donating partner for DIET (Shrestha et al., 2013; Rotaru et al., 2014a; Ueki et al., 2018) have suggested that c-type cytochromes and electrically conductive pili [e-pili] (Lovley, 2017a) facilitate electron transport from *G. metallireducens* to the electron accepting partner.

Outer-surface *c*-type cytochromes and e-pili are also involved in electron uptake by *G. sulfurreducens* when it is the electron-accepting partner in DIET-based co-cultures with *G. metallireducens* (Summers et al., 2010; Shrestha et al., 2013; Ueki et al., 2018). However, *Methanosarcina barkeri* and *Methanothrix* (formerly *Methanosaeta*) *harundinacea*, the only methanogens definitively shown to participate in DIET (Rotaru et al., 2014a,b), do not possess outer-surface *c*-type cytochromes or e-pili. Their outer surface electrical contacts for DIET are unknown.

The basic physiology and biochemistry of *M. barkeri* are much better understood than for *Mt. harundinacea* (Thauer et al., 2008; Gonnerman et al., 2013; Welte and Deppenmeier, 2014; Boone and Mah, 2015; Kulkarni et al., 2018; Mand et al., 2018). This makes *M. barkeri* the organism of choice for initial DIET mechanistic studies. Another advantage is that methods are available for genetic manipulation of *M. barkeri* (Kohler and Metcalf, 2012), but not *Mt. harundinacea*. However, one caveat for the study of DIET is that *M. barkeri* mutants have been previously constructed in a strain adapted to grow in high salt concentrations to prevent cell aggregation (Kohler and Metcalf, 2012). *G. metallireducens*, the only known electron-donating partner for *M. barkeri*, has yet to be adapted to grow at such high salt conditions.

Thus, at least at present, alternative approaches to evaluating the physiology of *M. barkeri* during DIET are required. Comparing the transcriptome of cells grown via DIET versus cells grown via HIT clearly reflected differences in electron uptake mechanisms in studies in which *G. sulfurreducens* functioned as the electron-accepting partner (Shrestha et al., 2013). *G. sulfurreducens* was grown by DIET with *G. metallireducens* as the electron-donating partner, or by HIT in co-culture with *Pelobacter carbinolicus* a microorganism closely related to *G. metallireducens*, but which is incapable of DIET (Shrestha et al., 2013). The *G. sulfurreducens* transcriptome demonstrated that cells were poised for growth on H_2_ when *G. sulfurreducens* was grown with *P. carbinolicus* and expressed genes for outer-surface proteins involved in direct uptake of electrons during DIET-based growth with *G. metallireducens* (Shrestha et al., 2013). *M. barkeri* can also be grown in co-culture with either *G. metallireducens* or *P. carbinolicus* (Rotaru et al., 2014a), providing an opportunity to compare *M. barkeri* gene expression patterns during growth via DIET and HIT.

**FIGURE 1:**
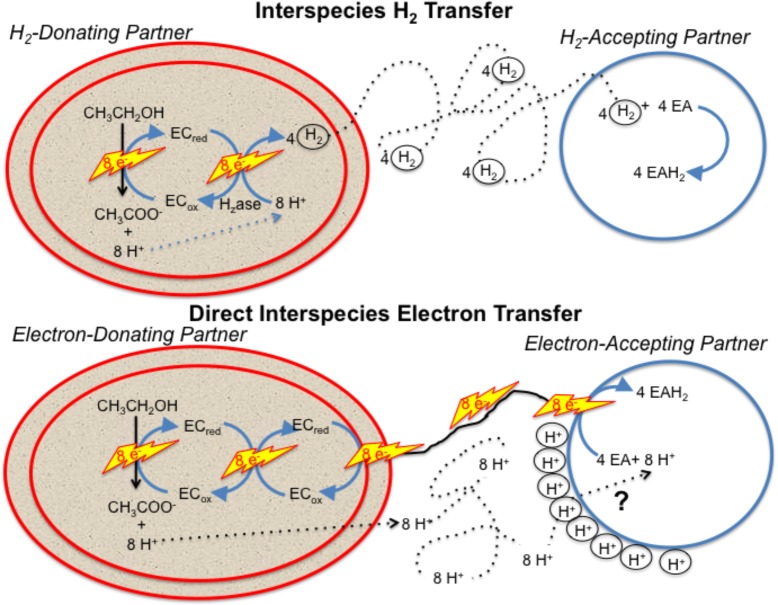
Generalized model for electron and proton flux during hydrogen interspecies electron transfer (HIT) and direct interspecies electron transfer (DIET) with growth on ethanol as an example. H_2_ diffusion shuttles both electrons and protons between cells and carries both electrons and protons into the cell when cytoplasmic electron acceptors are reduced. In contrast, electron and protons are transported by different mechanisms during DIET. Electron transfer is direct, through e-pili and other electrical contacts. Protons move by diffusion creating a positive proton pressure outside the cell. A mechanism for proton translocation into the cell is required for charge balance in the cytoplasm when cytoplasmic electron acceptors (EA) are reduced and to prevent acidification of the external space between cells. EC: electron carrier.

Any model describing how the electron-accepting partner utilizes electrons derived from DIET must account for the uncoupling of the routes for interspecies electron and proton flux (Figure 1). e-Pili only transport electrons. Protons move between DIET partners by diffusion. This uncoupled transport of electrons and protons is in stark contrast to HIT in which H_2_ simultaneously transports both electrons and protons as the H_2_ diffuses between the two partners. When the H_2_ is oxidized in the cytoplasm with electron transfer to an electron acceptor, protons are also released and are immediately available to balance the negative charge transferred to the electron acceptor. This maintains charge balance within the cell (Figure 1). In contrast, in DIET, e-pili and associated electron transport proteins deliver electrons to cytoplasmic electron acceptors. Protons have to be translocated into the cytoplasm for charge balance (Figure 1). This proton consumption also prevents acidification of the extracellular matrix of the DIET aggregates. Thus, proposed mechanisms for electron uptake during DIET need to include an explanation for how protons are translocated into the cytoplasm of the electron-accepting partner.

Here we report transcriptomic data from *M. barkeri* grown via DIET and HIT. The results suggest a mechanism for *M. barkeri* to utilize electrons and protons, derived from the electron-donating partner during DIET, to conserve energy to support growth from the reduction of carbon dioxide to methane.

## Materials and Methods

### Co-culture Incubation and mRNA Extraction

Triplicate replicates of co-cultures of *G. metallireducens/M. barkeri* and *P. carbinolicus/M. barkeri* were grown under strict anaerobic conditions as previously described (Rotaru et al., 2014a). Cultures were harvested during the exponential phase of growth and mRNA was isolated as previously described (Shrestha et al., 2013).

### Illumina Sequencing and Assembly of Reads

Directional multiplex libraries were prepared with the ScriptSeq^TM^ v2 RNA-Seq library preparation kit (Epicentre). Single end sequencing was performed with a Hi-Seq 2000 platform at the Deep Sequencing Core Facility at the University of Massachusetts Medical School in Worcester, MA, United States.

All raw data generated by Illumina sequencing were quality checked by visualization of base quality scores and nucleotide distributions with FASTQC^1^. Initial raw non-filtered forward and reverse sequencing libraries contained an average of 3892089 ± 134932 reads that were ∼100 basepairs long. Sequences from all of the libraries were trimmed and filtered with trimmomatic (Bolger et al., 2014) with the sliding window approach set to trim bases with quality scores lower than 3, strings of 3+N’s, and reads with a mean quality score lower than 20. Bases were also cut from the start and end of reads that fell below a threshold quality of 3, and any reads smaller than 50 bp were eliminated from the library. These parameters yielded an average of 2732020 ± 217212 quality reads per RNA-Seq library. SortMeRNA (Kopylova et al., 2012) was then used to separate all ribosomal RNA (rRNA) reads from the libraries. Databases used by SortMeRNA to identify all rRNA sequences included Rfam 5.8S Eukarya, Rfam 5S Archaea/Bacteria, SILVA 16S Archaea, SILVA 16S Bacteria, SILVA 23S Bacteria, SILVA 18S Eukarya, and SILVA 28S Eukarya (Burge et al., 2013; Quast et al., 2013).

### Mapping of mRNA Reads

Trimmed and filtered mRNA reads from the triplicate samples for the two different co-culture conditions were mapped against the genome of *M. barkeri* strain MS DSM 800 downloaded from IMG/MER^2^. Mapped reads were normalized with the RPKM (reads assigned per kilobase of target per million mapped reads) method (Mortazavi et al., 2008; Klevebring et al., 2010) using ArrayStar software (DNAStar). Graphical analysis of reads from all three biological replicates for each condition demonstrated that the results were highly reproducible. Therefore, all reported values were obtained after merging and averaging replicates. Expression levels were considered significant only when the log_2_ RPKM value was higher than that of the median RPKM value.

Out of the 3,809 predicted protein-coding genes in the *M. barkeri* MS genome, 1,912 and 1,909 genes had expression levels that were higher than the median in DIET- and HIT-grown cells, respectively.

### Genome Data Analysis

Sequence data for all of the bacterial genomes was acquired from the U.S. Department of Energy Joint Genome Institute^3^ or from GenBank at the National Center for Biotechnology Information (NCBI)^4^. Initial analyses were done with analysis tools available on the Integrated Microbial Genomes (IMG) website (see text footnote 2). Some protein domains were identified with NCBI conserved domain search (Marchler-Bauer et al., 2015) and Pfam search (Finn et al., 2016) functions. Transmembrane helices were predicted with TMpred (Hofmann and Stoffel, 1993), TMHMM (Krogh et al., 2001), and HMMTOP (Tusnady and Simon, 2001) and signal peptides were identified with PSORTb v. 3.0.2 (Yu et al., 2010) and Signal P v. 4.1 (Petersen et al., 2011).

### Accession Number

Illumina sequence reads have been submitted to the NCBI database under project number PRJNA501858 and accession SAMN10346831-SAMN10346836.

## Results and Discussion

As previously described (Rotaru et al., 2014a) co-cultures of *G. metallireducens* and *M. barkeri* that were well-adapted for growth via DIET required ca. 25 days to metabolize the 20 mM ethanol provided as substrate whereas *P. carbinolicus/M. barkeri* co-cultures required ca. 15 days. Both *G. metallireducens* and *P. carbinolicus* metabolized ethanol to acetate with either the production of H_2_ (*P. carbinolicus*) or extracellular electron transfer (*G. metallireducens*). *M. barkeri* metabolized acetate in both co-cultures, but in the initial growth phases of the cultures acetate production was faster than consumption, resulting in an accumulation of acetate (Rotaru et al., 2014a).

### Transcriptome Reflects Faster Growth During HIT and Possible Greater Importance of Membrane and Outer-Surface Proteins During DIET

**FIGURE 2:**
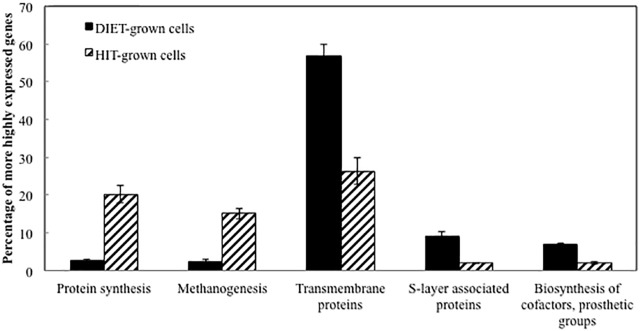
Comparison of relative expression of genes for different major classes of proteins during growth via DIET and HIT.

Transcript abundances for *M. barkeri* genes involved in amino acid biosynthesis, protein synthesis, and enzymes in the methane production pathways from both carbon dioxide and acetate were generally higher in the *P. carbinolicus/M. barkeri* co-cultures than in the *G. metallireducens/M. barkeri* co-cultures (Figure 2 and Supplementary Tables S1, S2). This is consistent with the faster growth of the *P. carbinolicus/M. barkeri* co-cultures. The highest proportion of genes that were more highly expressed during DIET-based growth were genes for proteins predicted to be associated with the membrane or cell surface (Figure 2).

Genes for all three functional *M. barkeri* hydrogenases [Ech, Frh, and Vht (Mand et al., 2018)] were more abundant during HIT-based growth (Table 1). However, the results suggest that it will not be possible to use hydrogenase gene transcript levels to diagnose whether *M. barkeri* is participating in HIT or DIET in microbial communities. The increase in hydrogenase gene expression in HIT-grown cells was comparable to the general increase in expression of genes for other methanogenesis enzymes, such as Mcr (Table 1 and Supplementary Table S2), suggesting that there was not a specific upregulation of hydrogenase genes in response to growth via HIT.

**Table 1:** Comparison of transcripts from genes coding for hydrogenase protein complexes (Ech, Frh, and Vht) and genes from the methyl coenzyme M reductase protein complex (Mcr) in *M. barkeri* cells growing via HIT in co-culture with *P. carbinolicus* or via DIET in co-culture with *G. metallireducens*.

			Fold up-regulated	log_2_	log_2_
Locus ID	Annotation	Gene	in HIT	RPKM DIET	RPKM HIT
Ga0072459_113104	Ech hydrogenase subunit F (ferredoxin)	*echF*	6.9	7.8*	10.2
Ga0072459_113103	Ech hydrogenase subunit E	*echE*	3.4	9.2	11.0
Ga0072459_113102	Ech hydrogenase subunit D	*echD*	8.7	7.9*	11.0
Ga0072459_113101	Ech hydrogenase subunit C	*echC*	3.5	8.8	10.6
Ga0072459_113100	Ech hydrogenase subunit B	*echB*	3.4	8.9	10.6
Ga0072459_113099	Ech hydrogenase subunit A, proton antiporter	*echA*	3.2	8.8	10.6
Ga0072459_113332	Coenzyme F420-reducing hydrogenase subunit beta	*frhB*	2.3	8.5	9.7
Ga0072459_113333	Coenzyme F420-reducing hydrogenase subunit gamma	*frhG*	2.5	8.1	9.5
Ga0072459_113335	Coenzyme F420-reducing hydrogenase subunit delta	*frhD*	3.6	7.2*	9.0
Ga0072459_113336	Coenzyme F420-reducing hydrogenase subunit alpha	*frhA*	2.0	8.5	9.5
Ga0072459_112833	Methanophenazine hydrogenase maturation protease	*vhtD*	ND	7.3*	7.2*
Ga0072459_112832	Methanophenazine-reducing hydrogenase, cytochrome B subunit	*vhtC*	4.6	7.5*	9.7
Ga0072459_112831	Methanophenazine-reducing hydrogenase large subunit	*vhtA*	2.2	8.7	9.9
Ga0072459_112830	Methanophenazine-reducing hydrogenase small subunit	*vhtG*	2.2	8.2	9.4
Ga0072459_1188	Methyl-coenzyme M reductase, alpha subunit	*mcrA*	3.5	10.1	11.9
Ga0072459_1187	Methyl-coenzyme M reductase, gamma subunit	*mcrG*	4.2	11.0	13.1
Ga0072459_1186	Methyl coenzyme M reductase, subunit C	*mcrC*	6.0	10.2	12.8
Ga0072459_1185	Methyl coenzyme M reductase, subunit D	*mcrD*	4.2	10.9	13.0
Ga0072459_1184	Methyl-coenzyme M reductase, beta subunit	*mcrB*	4.4	10.2	12.3

*The log_*2*_ RPKM median for HIT-grown *M. barkeri* cells was 7.5. The log_*2*_ RPKM median for DIET-grown *M. barkeri* cells was 7.9. ^∗^Transcripts with values below the log_*2*_ RPKM median. ND, no significant difference in transcription*.

**FIGURE 3:**
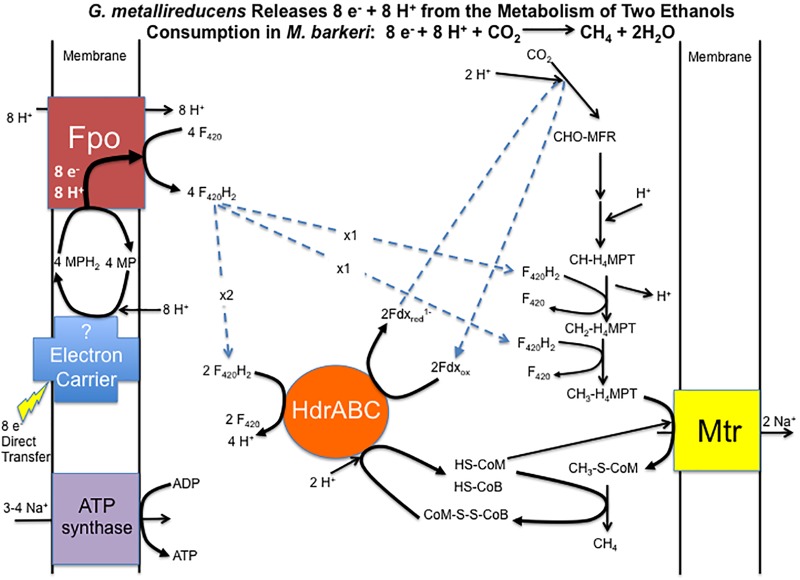
Model for electron and proton flux for carbon dioxide reduction to methane in *Methanosarcina barkeri* during DIET-based growth. Each two moles of ethanol oxidized to acetate by *G. metallireducens* releases eight electrons and eight protons. Electrons delivered to methanophenazine in the cell membrane are transferred to Fpo. Proton translocation drives Fpo-catalyzed reduction of F_420_ to F_420_H_2_. Half of the F_420_H_2_ produced serves as a reductant in the carbon dioxide reduction pathway. The remaining F_420_H_2_ is the electron donor for HdrABC, which reduces ferredoxin and CoM-S-S-CoB in an electron bifurcation reaction. The steps in carbon dioxide reduction, including the role of reduced ferredoxin, CoM-SH, and CoB-SH, as well as sodium pumping with Mtr, are as previously described (Thauer et al., 2008) for *M. barkeri.*

Considering that gene expression for many metabolic genes was generally lower in DIET-grown cells, any genes for which transcript abundance was higher during DIET, or even comparable to HIT-grown cells, are of considerable interest. In the following sections, genes with higher expression during growth on DIET are examined further. The results are placed in the context of a working model (Figure 3) for generating the reduced co-factors required for carbon dioxide reduction to methane (F_420_H_2_, reduced ferredoxin) while also providing a mechanism for CoM-S-S-CoB reduction and a chemiosmotic potential to provide energy to support DIET-based growth.

### Proton-Driven Reverse Electron Transport to Reduce F_420_ With Fpo

Transcripts for genes for most of the subunits for the membrane-bound F_420_H_2_ dehydrogenase, Fpo, were higher in DIET-grown cells (Table 2). Considering that transcripts for most genes for methanogenesis were more abundant in HIT-grown cells, these results suggest that Fpo plays a key role in electron transport for carbon dioxide reduction to methane during DIET. During methylotrophic methanogenesis Fpo oxidizes F_420_H_2_ with the reduction of methanophenazine in the membrane, coupled with vectorial proton translocation to the outside of the membrane (Welte and Deppenmeier, 2014; Kulkarni et al., 2018; Mand et al., 2018). However, under some conditions Fpo may catalyze the reverse reaction in which reduced methanophenazine serves as the electron donor for the reduction of F_420_ (Mand et al., 2018). In this direction, proton translocation through Fpo into the cytoplasm is required in order to make the reaction thermodynamically favorable.

**Table 2:** Comparison of transcripts from genes coding for subunits of Fpo dehydrogenase in *M. barkeri* cells growing via HIT in co-culture with *P. carbinolicus* or via DIET in co-culture with *G. metallireducens*.

			Fold up-regulated	log_2_	log_2_
Locus ID	Annotation	Gene	in DIET	RPKM DIET	RPKM HIT
Ga0072459_111718	F_420_H_2_ dehydrogenase subunit O	*fpoO*	2.9	7.5*	6.0*
Ga0072459_111719	F_420_H_2_ dehydrogenase subunit N	*fpoN*	2.4	8.2	6.9*
Ga0072459_111720	F_420_H_2_ dehydrogenase subunit M	*fpoM*	2.1	8.6	7.5*
Ga0072459_111721	F_420_H_2_ dehydrogenase subunit L	*fpoL*	1.5	8.5	7.9
Ga0072459_111722	F_420_H_2_ dehydrogenase subunit K	*fpoK*	3.7	8.3	6.4*
Ga0072459_111723	NADH dehydrogenase subunit J	*fpoJ*	2.4	9.7	8.5
Ga0072459_111724	F_420_H_2_ dehydrogenase subunit J	*fpoJ*	2.6	8.7	7.3*
Ga0072459_111725	F_420_H_2_ dehydrogenase subunit I	*fpoI*	1.7	7.6*	6.9*
Ga0072459_111726	F_420_H_2_ dehydrogenase subunit H	*fpoH*	2.1	9.3	8.2
Ga0072459_111727	F_420_H_2_ dehydrogenase subunit D	*fpoD*	1.7	8.3	7.6
Ga0072459_111728	F_420_H_2_ dehydrogenase subunit C	*fpoC*	1.5	7.2*	6.6*
Ga0072459_111729	F_420_H_2_ dehydrogenase subunit B	*fpoB*	2.1	8.0	6.9*
Ga0072459_111730	F_420_H_2_ dehydrogenase subunit A	*fpoA*	2.0	8.4	7.4*
Ga0072459_112975	F_420_H_2_ dehydrogenase subunit F	*fpoF*	1.0	9.0	9.1

*The log_*2*_ RPKM median for HIT-grown *M. barkeri* cells was 7.5. The log_*2*_ RPKM median for DIET-grown *M. barkeri* cells was 7.9. ^∗^Transcripts with values below the log_*2*_ RPKM median*.

Therefore, it is proposed that electrons derived from DIET reduce methanophenazine in the oxidized state (MP) to MPH_2_ and that MPH_2_ is the electron donor for Fpo to reduce F_420_ in the cytoplasm (Figure 3). A proton gradient to drive the reaction is available from the protons released into the extracellular matrix from *G. metallireducens* metabolism in direct proportion to electrons transported from *G. metallireducens* through e-pili. The proton flux through Fpo does not acidify the cytoplasm because an equivalent number of protons are consumed from the cytoplasm when MP is reduced to MPH_2_ (Figure 3). The protons required to produce MPH_2_ are transferred to F_420_ during the Fpo-catalyzed reaction MPH_2_ + F_420_ → MP + F_420_H_2_. In this way electron transfer through methanophenazine to F_420_ is achieved with charge balance.

### Possible Increased Methanophenazine Production to Support DIET

The proposed generation of F_420_H_2_ by Fpo with electrons derived from DIET requires an abundance of reduced methanophenazine (Figure 3). The pathway involved in biosynthesis of methanophenazine has not been identified, however, it is likely to resemble those of respiratory quinones because both have a polyprenyl side-chain connected to a redox-active moiety. In fact, studies have shown that a farnesylgeranyl pyrophosphate synthetase from the terpenoid backbone biosynthesis pathway is required for methanophenazine biosynthesis in *M. mazei* (Ogawa et al., 2010). Nine genes predicted to code for proteins involved in ubiquinone/menaquinone biosynthesis; six UbiE methyltransferase proteins, UbiA prenyltransferase, phenylacrylic acid decarboxylase (UbiD), and a ubiquinone biosynthesis protein (UbiB) were >2 fold more highly expressed in DIET-grown cells and another 11 putative ubiquinone biosynthesis genes were >1.5 fold up in DIET grown cells (Table 3). Given that *M. barkeri* does not contain ubiquinone or menaquinone, it seems likely that these genes code for enzymes involved in methanophenazine synthesis.

**Table 3:** Comparison of transcripts from genes coding for enzymes from the terpenoid backbone or terpenoid/quinone biosynthesis pathways in *M. barkeri* cells growing via DIET in co-culture with *G. metallireducens* or via HIT in co-culture with *P. carbinolicus*.

		Fold up-regulated	log_2_	log_2_
Locus ID	Annotation	in DIET	RPKM DIET	RPKM HIT
Ga0072459_112001	UbiE/COQ5 methyltransferase	4.0	9.0	7.0*
Ga0072459_11404	UbiE/COQ5 methyltransferase	3.1	8.2	6.5*
Ga0072459_113351	UbiE/COQ5 methyltransferase	2.8	8.2	6.8*
Ga0072459_11983	UbiE/COQ5 methyltransferase	2.5	8.3	7.0*
Ga0072459_111147	Ubiquinone biosynthesis protein	2.3	8.9	7.7
Ga0072459_11398	UbiE/COQ5 methyltransferase	2.3	7.9	6.7*
Ga0072459_11322	UbiE/COQ5 methyltransferase	2.1	8.7	7.6
Ga0072459_111453	UbiA prenyltransferase	2.0	8.0	6.9*
Ga0072459_11572	Phenylacrylic acid decarboxylase UbiD	2.0	7.7*	6.7*
Ga0072459_11988	UbiE/COQ5 methyltransferase	1.8	7.8*	6.9*
Ga0072459_113640	UbiE/COQ5 methyltransferase	1.8	8.2	7.4*
Ga0072459_112914	UbiA prenyltransferase	1.7	8.5	7.8
Ga0072459_111590	UbiE/COQ5 methyltransferase	1.7	7.8*	7.0*
Ga0072459_111148	UbiE/COQ5 methyltransferase	1.7	7.5*	6.8*
Ga0072459_112908	Demethylmenaquinone methyltransferase	1.7	7.3*	6.6*
Ga0072459_113090	UbiE/COQ5 methyltransferase	1.6	7.1*	6.4*
Ga0072459_112235	UbiA prenyltransferase	1.6	7.7*	7.0*
Ga0072459_113530	UbiE/COQ5 methyltransferase	1.5	7.5*	6.9*
Ga0072459_112514	UbiE/COQ5 methyltransferase	1.5	7.7*	7.1*
Ga0072459_113346	UbiE/COQ5 methyltransferase	1.5	7.5*	7.0*
Ga0072459_113347	UbiE/COQ5 methyltransferase	1.4	8.2	7.7
Ga0072459_11898	UbiA prenyltransferase	1.4	8.2	7.7
Ga0072459_11516	Isopentenyl phosphate kinase	1.3	8.8	8.4
Ga0072459_11638	Farnesylgeranyl pyrophosphate synthetase	1.2	7.4*	7.2*
Ga0072459_113679	UbiA prenyltransferase	1.2	8.4	8.2
Ga0072459_11517	Isopentenyl-diphosphate delta-isomerase	1.0	8.5	8.5
Ga0072459_11519	Geranylgeranyl-diphosphate synthase	0.7	8.1	8.6

*Negative values in the column “fold up-regulated in DIET” indicate that transcripts were more abundant in HIT-grown cells. The log_*2*_ RPKM median for HIT-grown *M. barkeri* cells was 7.5. The log_*2*_ RPKM median for DIET-grown *M. barkeri* cells was 7.9. ^∗^Transcripts with values below the log_*2*_ RPKM median*.

It has been calculated that the concentration of methanophenazine in membranes of *M. acetivorans* grown on methanol is sufficient to convert the membrane into an “electrically quantitized” conductive material (Duszenko and Buan, 2017). Methanophenazine concentrations in membranes of methanol-grown *M. barkeri* were too low for this effect (Duszenko and Buan, 2017). However, increased methanophenazine synthesis during growth via DIET might also yield an electrically quantitized membrane in *M. barkeri*, alleviating the need for redox-active proteins to aid in electron transport through the membrane during DIET.

### Generating Reduced Ferredoxin and Reducing CoM-S-S-CoB With HdrABC

In addition to F_420_H_2_, *M. barkeri* needs to generate reduced ferredoxin during DIET. It is required for the first step in carbon dioxide reduction to methane (Thauer et al., 2008). One of the few soluble protein complexes with higher gene transcript abundance during DIET is the heterodisulfide reductase HdrA1B1C1 (Table 4), suggesting that it is important for DIET. Transcript abundance for the genes for subunits of the homologous HdrA2B2C2 was more comparable to that during growth on HIT, with the transcripts for the genes of the A2 and B2 slightly higher during DIET and lower transcripts for the C2 subunit. When the general pattern of higher gene transcript abundance for soluble proteins in HIT-grown cells is considered, these results suggest that HdrA2B2C2 might also be important in DIET.

**Table 4:** Comparison of transcripts from genes coding for heterodisulfide reductase complexes HdrA1B1C1 and HdrA2B2C2 in *M. barkeri* cells growing via HIT in co-culture with *P. carbinolicus* or via DIET in co-culture with *G. metallireducens*.

Locus ID	Annotation	Gene	Fold up-regulated in DIET	log_2_ RPKM DIET	log_2_ RPKM HIT
Ga0072459_11778	Heterodisulfide reductase subunit A1	*hdrA1*	1.93	8.86	7.91
Ga0072459_11777	Heterodisulfide reductase subunit C1	*hdrC1*	2.04	9.32	8.29
Ga0072459_11776	Heterodisulfide reductase subunit B1	*hdrB1*	2.55	8.62	7.27*
Ga0072459_111651	Heterodisulfide reductase subunit A2	*hdrA2*	1.27	8.75	8.40
Ga0072459_113523	Heterodisulfide reductase subunit B2	*hdrB2*	1.25	8.63	8.31
Ga0072459_113524	Heterodisulfide reductase subunit C2	*hdrC2*	-2.05	6.80*	7.84
Ga0072459_113160	Heterodisulfide reductase subunit E	*hdrE*	-2.63	9.08	10.47
Ga0072459_113159	Heterodisulfide reductase subunit D	*hdrD1*	-3.22	7.94*	9.63
Ga0072459_11492	Heterodisulfide reductase subunit D2	*hdrD2*	2.05	7.80*	6.75*

*The log_2_ RPKM median for HIT grown *M. barkeri* cells was 7.5. The log_2_ RPKM median for DIET grown *M. barkeri* cells was 7.9. Negative values in the “Fold up in DIET” column indicate that genes were more highly transcribed in HIT-grown cells. ^∗^Transcripts with values below the log_*2*_ RPKM median*.

*In vitro* purified HdrA2B2C2 from *M. acetivorans* oxidizes F_420_H_2_ with the reduction of ferredoxin and CoB-S-S-CoM through flavin-based electron bifurcation (Yan et al., 2017). The phenotypes for various *Methanosarcina* mutants have suggested that HdrA1B1C1 can couple the oxidation of reduced ferredoxin with the reduction of both F_420_ and CoB-S-S-CoM (Buan and Metcalf, 2010; Gonnerman et al., 2013). However, this reaction has not been verified biochemically (Yan and Ferry, 2018) and the direction of electron flow for the HdrA1B1C1 complex could be similar to that demonstrated for the HdrA2B2C2 complex, especially under conditions in which there is substantial production of reduced F_420_ and limited routes for generating reduced ferredoxin. An electron bifurcation reaction in this direction would also be consistent with the electron bifurcation from flavin with the reduction of CoB-S-S-CoM and ferredoxin associated with the MvhADG/HdrABC complexes found in methanogens that specialize in growth with H_2_/CO_2_ (Kaster et al., 2011).

### The Completed Pathway for Energy Conservation During DIET

Therefore, it is proposed that half of the F_420_H_2_ generated with Fpo is the electron donor for HdrABC (one or both homologs) to produce reduced ferredoxin with the simultaneous reduction of CoM-S-S-CoB (Figure 3). In this way the coupled activity of Fpo- and HdrABC-catalyzed reactions deliver the eight moles of electrons derived from the oxidation of two moles of ethanol to each required step in the carbon dioxide reduction pathway (Figure 3).

As noted above, the Fpo-catalyzed reduction of F_420_ is proton balanced. Protons are released from F_420_H_2_ oxidation by HdrABC, but an equivalent number of protons are consumed in other reactions in the carbon dioxide reduction pathway (Figure 3). Thus, the model also balances proton flux.

The proposed model generates a chemiosmotic gradient to produce ATP through the activity of the Mtr complex that is known to pump sodium across the membrane during methyl transfer in the carbon dioxide reduction pathway (Thauer et al., 2008). There are two possibilities for ATP generation from the sodium gradient. Genes for both the A_1_A_0_ ATP synthase and the F_1_F_0_ ATP synthase were expressed during DIET (Supplementary Table S3). The available evidence suggests that both can translocate sodium (Schlegel and Muller, 2013). Genes for several components of the F_1_F_0_ ATP synthase were more highly expressed during growth on DIET and others were expressed at levels comparable to HIT-grown cells (Supplementary Table S3). This suggests that the F_1_F_0_ ATP synthase may play a more important role during growth on DIET, but at present there is not enough information on F_1_F_0_ ATP function in *M. barkeri* to speculate why.

### Transcriptomics Suggests Potential Outer Surface Electrical Contacts

A number of genes predicted to encode redox active proteins expected to be associated with the *M. barkeri* membrane and/or cell surface were more highly expressed in cells grown via DIET (Table 5). However, it is premature to speculate on their possible role in mediating electron transfer into the cell in the absence of further biochemical characterization to determine whether important characteristics, such as redox potential and cellular localization, are appropriate for proposed roles.

**Table 5:** Genes coding for putative transmembrane and/or surface associated electron transport proteins potentially involved in electron up-take during DIET.

				Evidence of	Fold	log_2_	log_2_	Redox	
		Signal	# Trans-membrane	surface	up-regulated	RPKM	RPKM	protein	
Locus ID	Annotation	peptide	helices	protein	in DIET	DIET	HIT	category	Localization
Ga0072459_111371	Cupredoxin domain protein	No	1	PS51257	7.3	10.4	7.5	Cupredoxin	Membrane
Ga0072459_113267	Cupredoxin	No	1	No	2.9	7.9	6.4*	Cupredoxin	Potentially extracellular
Ga0072459_113594	Cytochrome cd1-nitrite reductase-like, haem d1 domain	No	0	pfam08309	2.6	7.7*	6.4*	Cytochrome b/d	Potentially extracellular
Ga0072459_112903	Cytochrome bd-type quinol oxidase	No	9	No	2.1	7.7*	6.6*	Cytochrome b/d	Membrane
Ga0072459_11415	Cytochrome b5-like heme/steroid binding domain	No	1	No	2.0	7.8*	6.8*	Cytochrome b/d	Membrane
Ga0072459_113465	4Fe-4S ferredoxin-type, iron-sulfur binding domain	No	2	No	2.0	8.0	7.0*	Ferredoxin	Membrane
Ga0072459_11712	PQQ domain protein	No	0	PS51257	4.3	9.2	7.1*	Quinonprotein	Potentially extracellular
Ga0072459_111886	PQQ domain protein	No	2	No	2.8	8.6	7.1*	Quinonprotein	Potentially extracellular
Ga0072459_113595	PQQ domain protein	No	1	pfam08309	2.2	8.44	7.3*	Quinonprotein	Potentially extracellular
Ga0072459_111452	Secreted thioredoxin protein	Yes	0	PS51257	2.7	8.9	7.5	Thioredoxin	Extracellular

*These genes were at least two fold more highly expressed in *M. barkeri* cells grown via DIET than *M*. *barkeri* cells grown via HIT. PS51527: prokaryotic lipoprotein attachment site; pfam08309: LVIVD repeat found in bacterial and archaeal surface proteins. Cell localization predictions were made with PSORTb v3.0.2 software. ^∗^Transcripts with values below the log_*2*_ RPKM median (7.5 for HIT and 7.9 for DIET)*.

For example, a gene putatively encoding a membrane-bound protein with a cupredoxin domain (Ga0072459_111371) was highly expressed specifically during DIET (Table 5). The cupredoxins rusticyanin and sulfocyanin play important roles in electron transfer into cells of *Acidithiobacillus* and *Sulfolobus* species during Fe(II) and S^0^ oxidation (Komorowski and Schafer, 2001; Dennison, 2005) and thus might play a similar role in electron transport into *M. barkeri*. The mid-point potentials of known cupredoxins (150 to 680 mV) are more positive than that expected for an electron carrier involved in electron transport to methanophenazine [mid-point potential of -165 mV (Tietze et al., 2003)]. However, modifications in cupredoxin structure and environment may greatly influence their mid-point potential (Marshall et al., 2009) and thus a role in electron transport into the cell is conceivable.

In a similar manner, genes encoding proteins that putatively incorporate pyrroloquinoline quinone (PQQ) as a co-factor were highly expressed during growth via DIET (Table 5). Like rusticyanin and sulfocyanin, proteins with PQQ-binding domains are involved in electron transport into the cell during oxidation of Fe(II) or Mn(II) (Croal et al., 2007; Johnson and Tebo, 2008). The mid-point potential of proteins with PQQ domains (∼90–100 mV) is too positive to play a role in electron transfer to methanophenazine. However, genes for PQQ biosynthesis were not found in the *M. barkeri* genome. Thus, it is possible that these proteins with predicted PQQ domains may incorporate another co-factor. Methanophenazine is one possibility. Further analysis of these proteins and others with higher gene expression during DIET (Table 5) is warranted. The expression of genes for a number of soluble electron carriers/co-factors were higher in DIET-grown cells, further suggesting differences in electron flux during DIET (Supplementary Table S4), but more analysis will be required to evaluate their role/significance.

## Implications

The results suggest a pathway for electron and proton flux in *M. barkeri* during DIET that is significantly different than during HIT-based growth. The increased expression of genes for key components, including Fpo and HdrABC, and considerations of electron and proton transport during DIET, suggest an electron- and proton-balanced model in which the required electron donors are generated for each of the reductive steps of carbon dioxide reduction to methane while conserving energy to support growth (Figure 3). This model provides hypotheses that can be further evaluated experimentally with the appropriate *M. barkeri* mutants. However, as noted in the Introduction, this will require the discovery or development of an electron-donating strain capable of growing in the high salt medium that is used to generate *M. barkeri* mutants (Kohler and Metcalf, 2012). An alternative approach might be to adapt *M. barkeri* mutants to lower salt conditions, but this would require a time-consuming, labor-intensive adaption of each *M. barkeri* mutant strain with the risk of additional mutations arising during the adaption process.

The DIET transcriptome did not conclusively identify electrical contacts for DIET beyond the cell membrane. One potential reason for this is that *M. barkeri* might constitutively express these contacts. It is difficult to envision how *Geobacter* or other electron-donating partners could make electrical contacts with the outer surface of *M. barkeri* unless those contacts were expressed in advance of the initial *Geobacter-M. barkeri* electrical interaction. *M. barkeri*’s low affinity for H_2_ makes it a poor competitor for H_2_ in many environments (Thauer et al., 2008). Constitutive production of outer surface electrical contacts could poise *M. barkeri* for DIET and provide a competitive advantage in utilizing this alternative source of electrons for carbon dioxide reduction.

Elucidating the role of *Methanosarcina* species in DIET in complex natural environments is complicated by the possibility that H_2_ must also be considered as a potential electron donor for carbon dioxide reduction (Holmes et al., 2017). The differences in gene expression patterns between DIET- and HIT-grown cells suggest that metatranscriptional analysis is a route to better characterize the extent to which *Methanosarcina* are involved in DIET. It has been suggested that *M. barkeri* as well as other methanogens, can directly accept electrons from other extracellular sources such as electrodes, conductive carbon materials, and metals, but it has been difficult to rule out the possibility that H_2_ might be an intermediary electron carrier (Cheng and Call, 2016; Blasco-Gomez et al., 2017; Lovley, 2017b,c). The finding that transcriptome patterns in cells directly accepting electrons from an external source differ substantially from cells utilizing H_2_ as an electron donor suggests that the transcriptomic analysis approach described here could also help resolve this question.

## Author Contributions

DH, A-ER, PS, and DL conceived the study. A-ER grew the co-cultures. PS extracted and processed the nucleic acids for sequences. DH re-annotated the genome as necessary and analyzed the transcriptome data. DH and DL wrote the initial version of the manuscript. All authors made important modifications and additions.

## Conflict of Interest Statement

The authors declare that the research was conducted in the absence of any commercial or financial relationships that could be construed as a potential conflict of interest.

## Footnotes

1 http://www.bioinformatics.babraham.ac.uk/projects/fastqc/

2 http://img.jgi.doe.gov

3 http://www.jgi.doe.gov

4 http://www.ncbi.nlm.nih.gov
