# Supplementary material for: Electron and Proton Flux for Carbon Dioxide Reduction in Methanosarcina barkeri During Direct Interspecies Electron Transfer
Source: Front Microbiol. 2018 Dec 13;9:3109. doi: 10.3389/fmicb.2018.03109 (PMC6315138; doi:10.3389/fmicb.2018.03109)
Supplement: Supplementary file 3 [file Table_3.DOCX]

Supplementary Table S3. Transcripts from genes coding for various ATP synthase proteins in *Methanosarcina barkeri* cells growing via HIT in co-culture with *Pelobacter carbinolicus* or via DIET in co-culture with *Geobacter metallireducens*. The RPKM log_2_ median for HIT-grown *M. barkeri* cells was 7.5. The RPKM log_2_ median for DIET-grown *M. barkeri* cells was 7.9. Negative values in the column titled “Fold up-regulated in HIT” indicate that transcripts were more significantly transcribed in DIET-grown cells.

*Transcripts with values below the log_2_ RPKM median

| Locus ID | Annotation | log_2_ RPKM DIET | log_2_ RPKM HIT | Fold up-regulated in HIT |
| --- | --- | --- | --- | --- |
| Ga0072459_11628 | A-type ATP synthase subunit D | 8.1 | 10.8 | 6.5 |
| Ga0072459_11629 | A-type ATP synthase subunit B | 8.3 | 10.3 | 4.0 |
| Ga0072459_11630 | A-type ATP synthase subunit A | 8.6 | 10.4 | 3.5 |
| Ga0072459_11631 | A-type ATP synthase subunit F | 8.6 | 10.5 | 3.8 |
| Ga0072459_11632 | A-type ATP synthase subunit C | 9.4 | 11.7 | 4.7 |
| Ga0072459_11633 | A-type ATP synthase subunit E | 11.6 | 9.2 | 5.4 |
| Ga0072459_11634 | A-type ATP synthase subunit K | 7.7* | 10.2 | 5.8 |
| Ga0072459_11635 | A-type ATP synthase subunit I | 9.0 | 11.9 | 7.5 |
| Ga0072459_11636 | A-type ATP synthase subunit H | 9.4 | 11.7 | 4.9 |
| Ga0072459_112348 | F-type ATP synthase, subunit gamma | 8.6 | 7.6 | -2.0 |
| Ga0072459_112349 | F-type ATP synthase subunit alpha | 8.1 | 8.0 | ND |
| Ga0072459_112350 | F-type ATP synthase F0 subcomplex B subunit | 8.5 | 8.3 | ND |
| Ga0072459_112351 | F-type ATP synthase F0 subcomplex C subunit, | 9.8 | 7.9 | -3.8 |
| Ga0072459_112352 | F-type ATP synthase F0 subcomplex A subunit | 8.6 | 8.2 | ND |
| Ga0072459_112353 | F-type ATP synthase, Methanosarcina type, subunit 2 | 8.6 | 8.2 | ND |
| Ga0072459_112354 | F-type ATP synthase, subunit 1 | 8.1 | 7.8 | ND |
| Ga0072459_112355 | F-type ATP synthase, subunit epsilon | 8.2 | 7.2* | -2.0 |
| Ga0072459_112356 | F-type ATPase subunit beta | 8.1 | 8.0 | ND |
