# Supplementary material for: Electron and Proton Flux for Carbon Dioxide Reduction in Methanosarcina barkeri During Direct Interspecies Electron Transfer
Source: Front Microbiol. 2018 Dec 13;9:3109. doi: 10.3389/fmicb.2018.03109 (PMC6315138; doi:10.3389/fmicb.2018.03109)
Supplement: Supplementary file 4 [file Table_4.DOCX]

Supplementary Table S4. Comparison of transcripts from genes coding for cytoplasmic electron transport proteins that were more highly transcribed in DIET-grown *Methanosarcina barkeri* cells than HIT-grown *M. barkeri* cells. The RPKM log_2_ median for HIT-grown *M. barkeri* cells was 7.5. The RPKM log_2_ median for DIET-grown *M. barkeri* cells was 7.9.

*Transcripts with values below the log_2_ RPKM median

| Locus ID | Annotation | Fold up-regulated in DIET | log_2_ RPKM DIET | log_2_ RPKM HIT |
| --- | --- | --- | --- | --- |
| Ga0072459_112200 | Protein with ferredoxin and flavodoxin domains | 2.37 | 7.7* | 6.1* |
| Ga0072459_111429 | Protein with ferredoxin and flavodoxin domains | 2.93 | 8.1 | 6.5* |
| Ga0072459_111731 | Putative Nitrite/sulfite reductase ferredoxin domain protein | 5.77 | 9.6 | 7.1* |
| Ga0072459_113161 | Putative Nitrite/sulfite reductase ferredoxin domain protein | 3.49 | 9.1 | 7.3* |
| Ga0072459_11364 | Uncharacterized protein with ferredoxin domain | 2.91 | 8.3 | 6.8* |
| Ga0072459_111961 | Uncharacterized protein with ferredoxin domain | 2.96 | 9.0 | 7.4* |
| Ga0072459_111681 | NADH:flavin oxidoreductase | 1.95 | 7.8* | 6.8* |
| Ga0072459_11275 | FldA-like Flavodoxin | 5.49 | 9.5 | 7.1* |
| Ga0072459_11828 | NAD(P)H:quinone oxidoreductase, type IV/flavoprotein-like WrbA | 3.18 | 9.0 | 7.4* |
| Ga0072459_113576 | NAD(P)H:quinone oxidoreductase, type IV/flavoprotein-like WrbA | 2.37 | 8.4 | 7.2* |
| Ga0072459_112466 | NAD(P)H:quinone oxidoreductase, type IV/flavoprotein-like WrbA | 1.90 | 8.3 | 7.4* |
| Ga0072459_113575 | NADH-FMN oxidoreductase RutF | 2.13 | 8.8 | 7.7 |
| Ga0072459_112981 | archaeoflavoprotein AfpA | 2.40 | 8.9 | 7.4* |
| Ga0072459_111785 | NADP oxidoreductase; aldo/keto reductase | 2.11 | 8.0 | 7.0* |
| Ga0072459_112971 | Cytochrome b5-like heme/steroid binding domain protein | 3.10 | 10.3 | 8.7 |
